# Next-Generation Sequencing and Bioinformatics Consortium Approach to Genomic Surveillance

**DOI:** 10.3201/eid3014.240306

**Published:** 2024-11

**Authors:** Lindsay C. Morton, Nazia Rahman, Kimberly A. Bishop-Lilly

**Affiliations:** Cherokee Nation Strategic Programs, Tulsa, Oklahoma, USA (L.C. Morton, N. Rahman); Global Emerging Infections Surveillance Branch, Silver Spring, Maryland, USA (L.C. Morton, N. Rahman); Naval Medical Research Command Biological Defense Research Directorate, Fort Detrick, Maryland, USA (K.A. Bishop-Lilly)

**Keywords:** Genomic surveillance. Global Emerging Infections Surveillance, GEIS, Next-Generation Sequencing and Bioinformatics Consortium, sequencing, bioinformatics, consortium

## Abstract

Genomic surveillance programs benefit greatly from a network of committed, well-supported laboratories that conduct ongoing surveillance activities for pathogens of public health importance. The experiences of the Global Emerging Infections Surveillance program provide insights for building and maintaining genomic surveillance capabilities for public health and pandemic preparedness and response. To meet the needs of US Department of Defense and the Military Health System to use genomics to monitor pathogens of military and public health importance, Global Emerging Infections Surveillance convened a consortium of experts in genome sequencing, bioinformatics, and genomic epidemiology. The experts developed a 3-tiered framework for building and maintaining next-generation sequencing and bioinformatics capabilities for genomic surveillance within the Department of Defense. The consortium strategy was developed before the COVID-19 pandemic, leading to a network prepared to respond with existing resources and expand as new funding became available.

The Global Emerging Infections Surveillance (GEIS) program, established in 1997 under the US Department of Defense (DoD), is responsible for distributing funding and monitoring projects to support global surveillance for infectious diseases with pandemic potential and of importance to the US military health system (*1*). DoD medical research laboratories were among the earliest adopters of next-generation sequencing (NGS) technologies—genomic sequencing technologies developed after Sanger sequencing, such as 454 (*2*)—to enhance surveillance for infectious diseases of military and global health importance (*3*,*4*). NGS approaches can provide higher-throughput testing (*5*), identification of and creation of new taxa for novel or unexpected organisms (*6*), and advanced molecular characterization such as genetic investigation of emerging pathogens; for example, in Bennett et al. (*7*). Some early examples of GEIS-funded surveillance programs using sequencing for pathogen surveillance and outbreak investigations are the DoD Global Respiratory Surveillance Program (*8*) and the Multidrug-Resistant Organism Repository and Surveillance Network (*9*,*10*). Over time, GEIS funds were used to purchase and maintain sequencing platforms, bioinformatics software, and computational infrastructure for genomic data collection and analysis. As NGS technologies became more mature and commonly available, a growing portion of the GEIS portfolio contained sequencing and bioinformatics work, necessitating better coordination to set surveillance priorities and develop and implement the strategic direction of pathogen genomic sequencing efforts.

In 2017, GEIS created a consortium of NGS laboratories to better administer limited resources and coordinate NGS and bioinformatics activities funded by GEIS. The primary purpose of the newly established consortium was to develop a sustainable and reliable laboratory network capable of fully using sequencing technologies for infectious disease surveillance and epidemic response activities. In the first iteration of the GEIS NGSBC (Next-Generation Sequencing and Bioinformatics Consortium) Strategic Plan, the Consortium leadership made programmatic recommendations for building and maintaining pathogen genomic surveillance capabilities within the DoD. The recommendations included designated partners providing technical support and training, as well as close communication among participating laboratories and stakeholders to build demonstrable competencies in genomic surveillance and technical aspects of sequencing and bioinformatics. Because of prior global investments in metagenomics and pathogen discovery by biodefense initiatives, the initial interest from DoD leadership was in standardizing metagenomic sequencing workflows for clinical samples of unknown etiology (*11*). However, the surveillance portfolios and limited bioinformatics infrastructure at overseas DoD laboratories led to prioritizing high-quality viral genomes for pathogens with pandemic potential, such as influenza viruses and arboviruses.

## Tiered Framework for Building and Maintaining Pathogen Sequencing and Bioinformatics Capabilities within the DoD

By 2019, the Consortium had developed a 3-tiered framework to support a series of layered assets and associated sequencing and bioinformatics capabilities within the DoD (*12*). Each sequencing laboratory has different needs for protocols, instrumentation, and personnel based on the physical location and the primary mission of the sequencing laboratory (e.g., clinical, medical research, or public health). Tier 1 represents the laboratories with the smallest footprint, flexibility, and most field-forward capabilities; tiers 2 and 3 scale up in laboratory complexity, size, and range of capabilities to account for operational needs and logistical needs (Figure). When designing the 3-tiered framework for sequencing laboratory capabilities, the Consortium assumed that companies and specific technologies change over time, so assets are typically grouped by sample and data throughput and laboratory space requirements (i.e., footprint). Today, the primary sequencers in use by GEIS partner laboratories include technologies from Illumina (https://www.illumina.com) and Oxford Nanopore Technologies (ONT; https://nanoporetech.com), which sell a range of devices that use different chemistries and vary in size, cost, and throughput (*3*,*13*,*14*). Genomic surveillance programs should be flexible enough to adopt rapidly improving sequencing technologies and bioinformatics software. However, differences in the speed of incorporation of newer technologies into existing sample sequencing workflows can cause substantial variation in methods among laboratories of the same tier.

**Figure:**
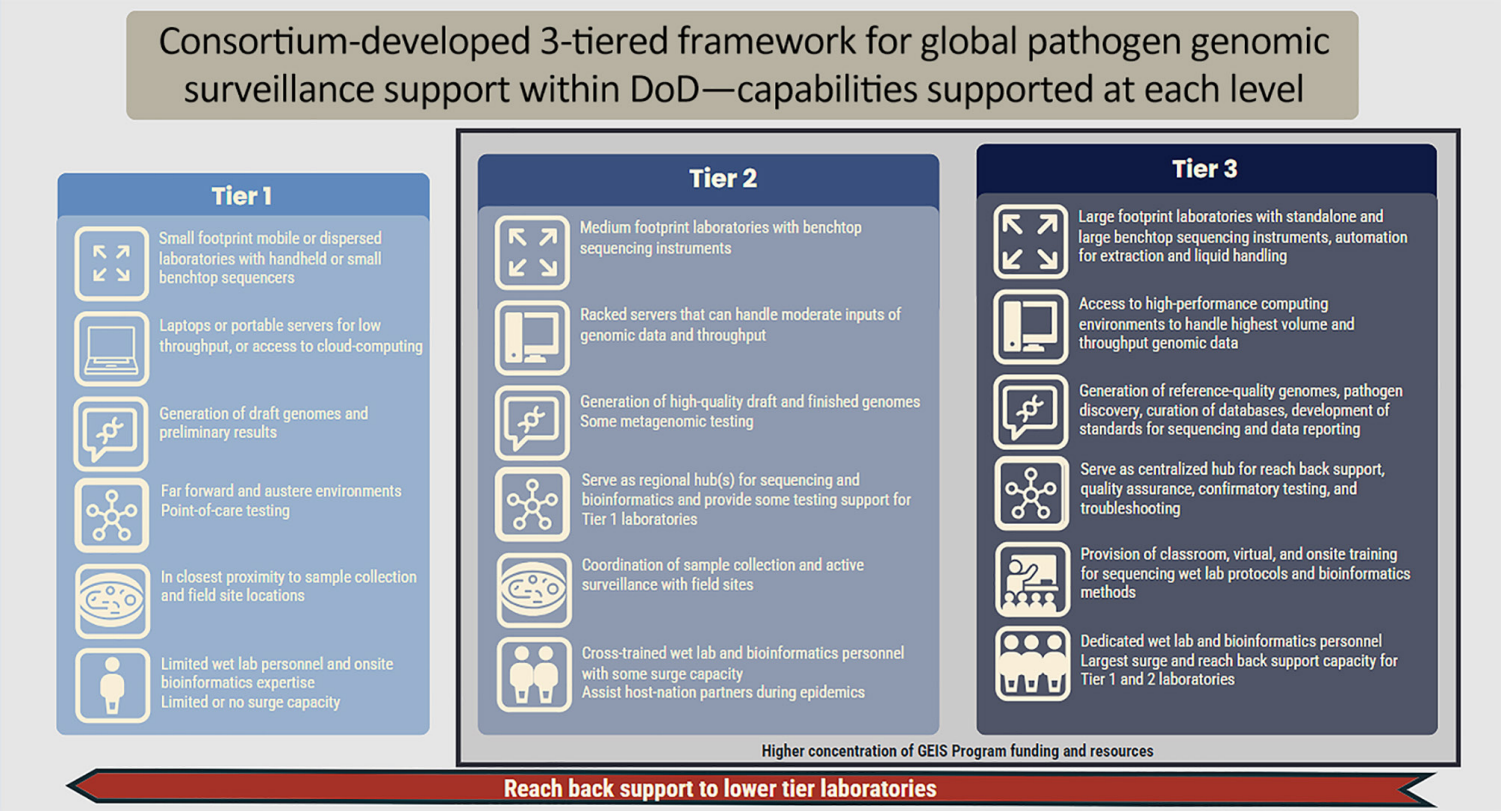
Tiered sequencing and bioinformatics capabilities for genomic surveillance within the US DoD. Modified from (*15*). DoD, Department of Defense; GEIS, Global Emerging Infections Surveillance.

### Tier 1

Tier 1 laboratories are in closest proximity to where samples are often collected. They are usually field sites, austere or forward operating environments, and places where point-of-care testing is conducted. Tier 1 laboratories have a small physical and technological footprint and are located mostly outside of the continental United States. The laboratories can quickly provide a preliminary result, such as pathogen identification, but typically will not perform deeper pathogen characterization. However, as sequencing methods and computational infrastructure become easier to implement in small or mobile laboratories, more characterization may occur at lower tiers. Tier 1 laboratories primarily use small hand-held or benchtop sequencers such as the ONT MinION or the Illumina MiniSeq and iSeq. Those platforms are most suitable for low-complexity samples, low throughput, and samples with previously suspected etiologic agents. In the laboratories, sequencing data are typically analyzed by using laptops and cloud-based bioinformatics. Regardless of throughput, tier 1 laboratories have lower sample transit time, enabling them to provide critical rapid responses to the GEIS network. Tier 1 partners also triage and select samples for additional analyses. Sequencing data may be produced at tier 1 and then after preliminary analyses sent back to tier 2 or 3 partners for further characterization. Historically, tier 1 laboratories have not been a GEIS NGSBC priority but are of high interest to the Armed Services, biodefense, and organizations with investments in mobile testing units.

### Tier 2

Tier 2 laboratories have an intermediate footprint, conducting NGS and bioinformatics farther from sample collection than tier 1 while maintaining the capability to serve as regional training and sequencing support centers. Wherever possible, a train-the-trainer approach is taken, in which tier 2 laboratories receive training via tier 3 laboratories to provide regional training and reach back to tier 1 (*15*,*16*). In addition to pathogen identification, tier 2 laboratories can conduct strain-level identification for viruses, bacteria, and some eukaryotes, with some degree of genetic characterization, including identification of virulence or antimicrobial resistance determinants. Most laboratories can perform agnostic sequencing on samples with limited metadata. Tier 2 laboratories process larger batches of samples with higher depth and breadth of coverage than tier 1 laboratories, which enables sequencing and analysis of more complex, metagenomic samples. The increased throughput typically involves the mid-sized Illumina platforms such as Illumina MiSeq or NextSeq for short-read sequencing, and ONT devices such as MinION or GridION for long-read sequencing. To support bioinformatics, tier 2 laboratories usually have >1 racked servers in addition to standalone workstations, rather than relying solely on laptops, cloud-based analytics, or both. Personnel are often cross-trained between the wet laboratory (nucleic acid extraction and sequencing) and bioinformatics methods. The NGSBC has focused on addressing gaps in training or equipment and on strengthening support to tier 2 laboratories.

### Tier 3

Tier 3 laboratories provide network-wide training and technical assistance while simultaneously running routine sequencing and bioinformatic analyses of samples collected throughout the GEIS network. The tier 3 laboratories located in the National Capital Region, the Naval Medical Research Command Biological Defense Research Directorate, the US Army Medical Research Institute of Infectious Diseases, and the Walter Reed Army Institute of Research collaboratively serve as core laboratories within NGSBC. The core laboratories assist the other NGSBC laboratories with gap assessments, development of curricula, training, coordination, and other forms of reach-back support (*15*). Tier 3 laboratories have the largest physical and technological footprints and have various combinations of short- and long-read sequencers, from ONT devices like GridION and PromethION or PacBio (Pacific Biosciences of California, https://www.pacb.com) to larger Illumina platforms such as NovaSeq. They also have a much larger repertoire of ancillary equipment, such as various machines for automated sample preparation, quality control assessments, or both. In tier 3 laboratories, high-performance computational resources are required to accompany the higher throughput of the sequencers in terms of depth of coverage of a given sample and the higher number of samples, the regular cadence of analyses, and the increased complexity inherent to advanced characterization. The level of throughput requires petabytes of data storage. In addition to strain-level identification, tier 3 laboratories routinely perform advanced genetic characterization including in silico genome closure, pathogen discovery, and phylogenetics. In tier 3 laboratories, personnel are typically dedicated to singular functions of wet laboratory or bioinformatic analysis. In comparison, personnel at tier 1 and tier 2 laboratories often perform laboratory and bioinformatics functions.

## Preparedness for Pandemic Response

After SARS-CoV-2 emerged in late 2019, GEIS was quickly recognized as the central coordinator for DoD COVID-19 genomic surveillance activities, leading to supplemental funding for domestic and overseas response efforts (*17*). Within the DoD, NGSBC tier 3 laboratories were involved in the early development, evaluation, and dissemination of standardized testing and sequencing protocols, bioinformatics workflows, and data reporting for COVID-19 (*18*). Outside the United States, several tier 2 laboratories with previous viral pathogen sequencing expertise were able to routinely sequence SARS-CoV-2 locally (*16*,*19*,*20*). GEIS also supported expanded sequencing capabilities and surge capacity in several laboratories in the Pacific (e.g., Japan, Hawaii, and the Philippines). As new SARS-CoV-2 variants emerged, focus shifted toward scaling up sequencing throughput and monitoring for changes potentially affecting medical countermeasures.

SARS-CoV-2 genomic surveillance data were used to directly inform public health mitigation efforts and deployment of medical countermeasures. GEIS partners developed and tested early sequencing protocols that can provide greater fidelity to answer questions such as whether persons were re-infected or their illness had recrudesced (*21*). Laboratories also developed novel bioinformatics methods to quickly scan for emerging intrahost SARS-CoV-2 variants from huge volumes of raw sequencing data (*22*). During the pandemic response, DoD public health and research groups demonstrated that genomics and phylogenetics could be used to understand the transmission of SARS-CoV-2 in military-specific settings, such as recruit or trainee depots and military installations (*23*,*24*), naval vessels (*25*), and overseas military locations (*26*–*28*). Genomic surveillance was also used to aid in naval outbreak investigations (*29*) and examine how well medical countermeasures and nonpharmaceutical interventions worked to slow or prevent SARS-CoV-2 spread in military populations (*23*,*30*).

## Improving the Post-COVID Pandemic Pathogen Sequencing Landscape

The US DoD 2023 Biodefense Posture Review urges the DoD to improve readiness and coordination for biosurveillance and bioincident response and specifically mentions increasing sequencing capabilities within the DoD (*31*). GEIS supports a network of partner laboratories ready to perform sequencing and bioinformatics for routine public health surveillance while being prepared to respond to the next pandemic or emerging pathogen. The Consortium continues to fill a gap in genomic surveillance coordination throughout the DoD. The annual program support for routine data and sample collection, sequencing exercises, procurement of NGSBI equipment and reagents, training, and retention of highly skilled personnel is essential for maintaining readiness. Support for pathogen genomic surveillance was a GEIS priority for many years before the COVID-19 pandemic. Several strategies used before and during the pandemic provided many benefits (Table), probably leading to success and overall improvements in the pathogen-sequencing landscape for DoD public health.

**Table:** Global Emerging Infections Surveillance program strategic activities for developing and maintaining genomic surveillance capabilities

Strategy	Description	Benefits
Consortium	Forming a group of NGS and bioinformatics subject-matter experts and well-established DoD laboratory partners led by an experienced program office	Ensures that genomic surveillance remains a priority for DoD
Tiered framework	Development of a 3-tiered strategic framework for public health investments in sequencing and bioinformatics within DoD	Prioritizes limited resources to maintain (or expand) genomic surveillance capabilities
Coordination meetings	Regular meetings for GEIS program, DoD, and non-DoD stakeholders	Provides better coordination across a diverse set of stakeholders
Funding	Leveraging diverse DoD and US government funding streams from public health to biodefense, biosecurity, and pandemic response(s)	Maintains genomic surveillance capabilities
Routine assessments	Continuous assessment of NGS and bioinformatics capabilities through a variety of assessment tools (e.g., site visits, structured/unstructured surveys, and proficiency testing exercises)	Provides “ground truth” or validation of capabilities
Appropriate interventions	Deployment of interventions (e.g., equipment, protocols, training, and reach back testing) to address identified gaps	Maintains genomic surveillance capabilities and equipment/personnel readiness
Tracking products	Tracking of genomic surveillance products (e.g., genomes produced/shared, publications, presentations, protocols developed, and technical assistance provided)	Demonstrates return on investments in genomic surveillance and potential effects on public health
Communication with leadership	Providing senior leadership with regular updates on findings and impacts from genomic surveillance	Improves public health decision-making

*DoD, Department of Defense; GEIS, Global Emerging Infections Surveillance; NGS, next-generation sequencing.

As public attention has waned and the urgency of the COVID-19 pandemic has ended, so has funding for many public health response activities. In the absence of the extra funding, maintenance of genomic surveillance readiness for infectious disease surveillance programs is in a precarious position. However, to maintain DoD genomic surveillance readiness for emerging biothreat response, funding for routine genomic surveillance programs such as seasonal respiratory pathogens or antimicrobial resistance is critical. In addition to funding, a reliable supply of samples for sequencing must be available, along with consistent, well-organized sample metadata that aid interpretation and analysis. The routine genomic surveillance activities that occur during the intermission between epidemics prevent erosion of capabilities and preserve existing infrastructure for the next pandemic response.

## Conclusions

During the past decade, GEIS used medical research and biodefense investments in sequencing technologies to build and maintain genomic surveillance capabilities within the DoD (*32*,*33*). Sustained progress has been possible because of a dedicated program office and a consortium of global partner laboratories with genomics expertise. The Consortium implemented a tiered framework to support NGS and bioinformatics capabilities within the network based on level of laboratory operations, with technical assistance and support to lower tiers (*15*). During the COVID-19 pandemic, Consortium laboratories responded quickly by adopting new protocols and expanding sequencing capabilities in support of DoD public health, medical countermeasure development and monitoring, and pandemic response. The GEIS program prioritization of genomic surveillance has led to a DoD network more prepared to respond to future epidemics and emerging pathogens.
